# Melanin particles isolated from the fungus *Fonsecaea pedrosoi* activates the human complement system

**DOI:** 10.1590/0074-02760180120

**Published:** 2018-06-25

**Authors:** Lysianne Pinto, Luiz Fernando Zmetek Granja, Mariana Amorim de Almeida, Daniela Sales Alviano, Maria Helena da Silva, Regina Ejzemberg, Sonia Rozental, Celuta Sales Alviano

**Affiliations:** 1 Universidade Federal do Rio de Janeiro, Instituto de Microbiologia Professor Paulo de Góes, Rio de Janeiro, RJ, Brasil; 2 Universidade Federal do Rio de Janeiro, Instituto de Macromoléculas Professora Eloisa Mano, Rio de Janeiro, RJ, Brasil; 3 Universidade Federal do Rio de Janeiro, Instituto de Biofísica Carlos Chagas Filho, Rio de Janeiro, RJ, Brasil

**Keywords:** Fonsecaea pedrosoi, fungus, complement system, melanin-ghost, melanin-particles

## Abstract

**BACKGROUND:**

Melanin production has been associated with virulence in various pathogenic fungi, including *Fonsecaea pedrosoi*, the major etiological agent for chromoblastomycosis, a subcutaneous fungal disease that occurs in South America.

**OBJECTIVE:**

The aim of this study was to evaluate the effects of acid-basic extracted *F. pedrosoi* melanin particles and fungal cell ghosts obtained by Novozym 234 treatment on their ability to activate the human complement system.

**METHODS:**

The ability of melanin particles and fungal cell ghosts to activate the human complement system was evaluated by complement consumption, immunofluorescence, and enzyme-linked immunosorbent assay (ELISA).

**FINDINGS:**

Unsensitised melanin particles and melanin ghosts presented complement consumption of 82.67 ± 2.08% and 96.04 ± 1.13%, respectively. Immunofluorescence assays revealed intense deposition of the C3 and C4 fragments on the surface of melanin particles and ghosts extracted from *F. pedrosoi*. Deposition of the C3, C4, and C5 fragments onto melanin samples and zymosan was confirmed by ELISA. Deposition of small amounts of C1q and C9 onto melanin samples and zymosan was detected by ELISA.

**CONCLUSION:**

*Fonsecaea pedrosoi* melanin particles and fungal cell ghosts activated the complement system mainly through an alternative pathway.

Chromoblastomycosis is a chronic progressive fungal infection of the mammalian skin and subcutaneous tissue caused by transcutaneous inoculation of a group of melanised fungi that belong to the *Dematiaceae* family (Torres-Guerrero et al. 2012). These fungi are saprophytes normally found in the mycelial form in tropical and sub-tropical soil and vegetation. Rounded fungal forms with transverse and longitudinal division are observed in the lesions and referred to as “sclerotic cells”. The etiological agent most commonly found in Brazil is *Fonsecaea pedrosoi*, which appears as thick-walled, brownish coloured cells because of the melanotic pigmentation in their cell walls (Correia et al. 2010).

*F. pedrosoi* constitutively produces melanin, a pigment and important virulence factor because of its anti-oxidative, thermostable, anti-radioactive, paramagnetic, and metal-binding properties (Cunha et al. 2010). Melanin production has been associated with virulence in various microorganisms and has been widely studied in several fungi such as *Aspergillus* spp, *Sporothrix schenckii*, and *Cryptococcus neoformans* (Romero-Martinez et al. 2000, Rosas et al. 2000, Gómez and Nosanchuck 2003). Fungi can produce melanin through several metabolic pathways. The most prevalent is the constitutive dihydroxynaphthalene pathway, found in *F. pedrosoi*, *Wangiella dermatitidis*, and *S. schenckii*. Another frequently described pathway is the dihydroxyphenylalanine (DOPA) pathway, based on melanin synthesis from the L-DOPA amino acid, and is present in *C. neoformans* (Nosanchuk et al. 2000, Cunha et al. 2005).

The complement system is an important part of innate immunity and plays an essential role in host defence against infectious agents and in inflammatory processes. It contains more than 30 proteins with enzymatic or binding properties and comprises multiple cell-surface receptors specific for fragments generated from the activation. These receptors are differentially expressed on the eosinophils, neutrophils, macrophages, monocytes, and B and T lymphocytes (Ricklin et al. 2010).

The complement system can be activated through classical pathways, typically involving an antigen-antibody complex, or by an alternative pathway, where the initial activation step occurs by spontaneous hydrolysis of the C3 molecule. C3 plays a central role in activation of the complement system, which is required for both classical and alternative complement activation pathways. C3 deficiencies increase the susceptibility to microorganism infections. Additionally, the complement can be activated by the lectin pathway, which involves activation through an interaction of mannose binding lectin or ficolins with carbohydrates expressed on microbial surfaces (Runza et al. 2008).

Numerous fungi such as *Aspergillus fumigatus*, *Candida albicans*, *C. neoformans*, and *Mucor* spp activate the complement system, mainly by the alternative pathway (Speth et al. 2008, Granja et al. 2010). Wünthrich et al. (2015) demonstrated that dectin-2 (and to a lesser extent dectin-1)-mediated recognition of *F. pedrosoi* is largely responsible for the development of Ag-specific Th17 cells, while Mincle appears to inhibit the development of this T-helper subset.

In the present study, we evaluated the role of melanin particles and fungal cell ghosts extracted from *F. pedrosoi* in activating the human complement system by complement consumption, immunofluorescence, and enzyme-linked immunosorbent assay (ELISA).

## MATERIALS AND METHODS

*Reagents and buffers* - The following buffers were used: PBS (10 mM phosphate, 15 mM saline, pH 7.2), PBS-Tween (PBS containing 0.05% of Tween 20), VBS (5 mM Veronal-buffered saline, pH 7.35-5 mM Veronal, 142 mM), GVB (VBS containing 0,1% gelatine), GVB^2+^ (GVB containing 0.15 mM CaCl_2_ and 1 mM MgCl_2_), GVB-EGTA-Mg^2+^ (GVB containing 10 mM EGTA (ethylene glycol-bis(b-aminoethyl ether)-N,N,N',N'-tetraacetic acid) and 10 mM MgCl_2_), and GVB-EDTA (GVB containing 10 mM EDTA), 10 mM sodium citrate-phosphate, pH 5.1. The following antibodies were used: rabbit serum anti-human C3c conjugated with fluorescein isothiocyanate and rabbit serum anti-human C4 purchased from Dako (Carpinteria, CA, USA) and goat serum anti-human C1q, goat serum anti-human C3c, rabbit serum anti-human C4, rabbit serum anti-human C5, rabbit serum anti-human C9, goat serum anti-rabbit IgG and rabbit serum anti-goat IgG conjugated with peroxidase and rabbit serum anti-sheep erythrocytes (haemolysin), and rabbit anti-guinea pig immunoglobulins (g-1 and/or g-2) purchased from Sigma Chemical Co. (St. Louis, MO, USA). All antibody dilutions were carried out according to the manufacturer's instructions.

*F. pedrosoi and zymosan* - A pathogenic strain *F. pedrosoi* (ATCC 46428, formerly 5VLP) isolated from a human case of chromoblastomycosis was used. Stock cultures were maintained on Sabouraud-dextrose-agar under mineral oil and kept at 4°C. For the experiments, a sample of *F. pedrosoi* was grown in Czapeck-Dox medium for 14 days at room temperature.

Zymosan A particles (Sigma Chemical Co.) were used as a positive control for complement activation. To loosen the mannan structures, zymosan particles were boiled for 30 min, washed with PBS, and resuspended in the same buffer (10 mg mL^-1^).

*Melanin particle extraction* - *F. pedrosoi* melanin particles were extracted from the mycelia using a Waring Blender with 0.5 M NaOH solution for 10 min. The mixture was kept under constant agitation for 24 h to completely extract the melanin. The sample was centrifuged at 5000 × *g* for 30 min and the supernatant was collected and treated with 6 M HCl until the pH reached 1.5. The resulting pigmented sample was washed several times with distilled water and lyophilised (Alviano et al. 1991).

*Melanin ghosts* - Melanin ghost isolation involved incubating the fungus with 1 M sorbitol in 0.1 M sodium citrate, pH 5.0. After washing in the same solution, the mycelia were treated with 10 mg Novozym 234 (Sigma Chemical Co.) in the same solution for 1 h at 37°C. The cells were separated by centrifugation at 1500 × *g* and resuspended in a 4 M guanidine isothiocyanate solution for 30 min at room temperature. The mixture was centrifuged and the pigmented material was transfer to 6 M HCl solution and heated to 100°C for 60 min. This material was extensively washed in distilled water, lyophilised, and analysed by light microscopy and infrared spectroscopy.

*Melanin sample spectrometry analyses* - In this study, a Frontier FT-IR spectrometer from PerkinElmer (Waltham, MA, USA) was used to compare the spectra of a commercial melanin sample obtained from *Sepia officinalis* (Sigma Chemical Co., M2649), melanin obtained from fungal supernatant, melanin in the ghost particle sample, and melanin treated with sodium hydroxide (NaOH). The spectra were obtained in transmission mode by the potassium bromide (KBr) pellet method. The previously dried samples were macerated together with KBr and then the mixture was pressed until pellets were formed. The KBr pellets were analysed from 4000 to 400 cm^-1^ with 4 cm^-1^ of resolution and 20 scans.

*Guinea pig immunisation* - Melanin particles were suspended (1 mg mL^-1^) in a sterile and apyrogenic saline solution. The suspension was mixed 1:1 (v v^-1^) with Freund's complete adjuvant (Sigma Chemical Co.). Intramuscular inoculation of two guinea pigs (*Cavia cobaya*) weighing approximately 1.2 and 1.3 kg each was carried out with 0.5 mL of the emulsified material. After 50 days, blood was collected and the obtained sera were aliquoted and stored at −80°C. One week later, both animals were administered another melanin intramuscular inoculation, but with Freund's incomplete adjuvant (Sigma Chemical Co.). After 21 days, blood was once more collected and the obtained sera were aliquoted and stored at −80°C. The antibody response was evaluated by agglutination with the melanin particles and melanin ghosts and observed under light microscopy. Pre-immune sera, collected before the immunisation process, were also maintained at −80°C. All animal experiments followed the guidelines of the local ethics committee.

*Human serum* - Peripheral blood was collected from healthy adult volunteers after obtaining informed consent. The sera were pooled and absorbed three times, 30 min each, with sheep red blood cells (1 × 10^9^ cells mL^-1^), all absorptions were carried out at 4°C. The sera were then absorbed three times with melanin particles (20 mg mL^-1^). This pool was used as the source of the human complement system. Serum was heated at 56°C for 30 min for studies requiring heat-inactivated serum. All serum samples were aliquoted and stored at −80°C.

*Opsonisation of sheep erythrocytes* - A sheep erythrocyte suspension (1 × 10^9^ cells mL^-1^) was opsonised by incubation with an equal volume of previously titrated haemolysin (erythrocyte-antibody, EA) at 37°C with agitation for 20 min, washed three times, and resuspended in GVB^2+^ to obtain a sample of 5 × 10^8^ cells mL^-1^.

*Complement activation* - Activation of the complement system by melanin (5 mg), melanin ghost (5 mg), and zymosan (5 mg) was carried out as follows. The samples were incubated with absorbed serum in either GVB^++^, GVB-Mg-EGTA, or GVB-EDTA. After 60 min at 37°C, the samples were centrifuged at 1500 × *g* under refrigeration and the supernatants were collected and residual complement was measured in a spectrophotometer (Beckman Coulter, Brea, CA, USA) at 540 nm using the haemolytic system (Granja et al. 2010). Melanin and melanin ghost samples sensitised with guinea pig anti-melanin antibodies (1/200 dilution, previously titrated) were subjected to the same activation process but without the addition of chelators. Zymosan was used as a positive control and serum alone, without cells, was used as a negative control. Haemolysis percentage was calculated by considering the positive and negative controls. The pellets were collected and washed three times with PBS-Tween for use in immunofluorescence assays and ELISA.

*Direct immunofluorescence assay* - After incubation and washing with PBS Tween, all samples and zymosan particles were washed with PBS. The particles were diluted 1/10 in PBS and incubated with fluorescein-conjugated rabbit anti-human C3c (dilution 1/25) for 30 min. The cells were washed and resuspended with PBS. One drop of each cell suspension was placed on a microscope slide, covered with a cover-slip, and examined under an epifluorescence microscope (Zeiss, Oberkochen, Germany).

*Indirect immunofluorescence assay* - After incubation and washing with PBS Tween, all samples and zymosan particles were washed with PBS. The particles were diluted 1/10 in PBS and incubated with rabbit anti-human C4 (dilution 1/25) for 30 min. Goat anti-rabbit secondary antibodies conjugated with fluorescein (1/50) were then added to react for 60 min at 37°C. The particles were washed and resuspended with PBS. One drop of each cell suspension was placed on a microscope slide, covered with a cover-slip, and examined under an epifluorescence microscope (Zeiss).

*ELISA* - To detect the deposition of the complement fragments (C1q, C3c, C4b, C5, and C9) on the surface of all samples, 96-well polystyrene plates (Corning, Inc., Corning, NY, USA) were coated with the particles obtained from complement activation (100 mL well^-1^). The plates were incubated for 1 h at 37°C, and then overnight at 4°C. The samples were washed with PBS-Tween 20 and blocking buffer (2% BSA in PBS) was added and incubated for 2 h at 37°C. The previously described primary antibodies were added to detect the fragments. The plates were incubated for 1 h at 37°C, and then washed three times with PBS-Tween 20. Secondary antibodies conjugated with peroxidase were added for 60 min at 37°C. The terminal complexes were detected after incubation for 30 min with substrate solution pH 5.0 (4 mL H_2_O_2_ and 4 mg OPD in 10 mL of 100 mM citric acid, 100 mM NaH_2_PO_4_). The absorbance was measured at 492 nm (SLT-Spectra, Tecan, Männedorf, Switzerland) after the reaction had been stopped with 2N H_2_SO_4_.

*Statistical analysis* - Complement activation, ELISA, and immunofluorescence assays were repeated at least three times. Complement consumption mean values were used for evaluation. Analysis of variance and two-tailed two-sample *t*-test were applied to compare results from each activation. Results were considered significant at P values of < 0.05 for a two-sided test.

## RESULTS

**Fig. 1:**
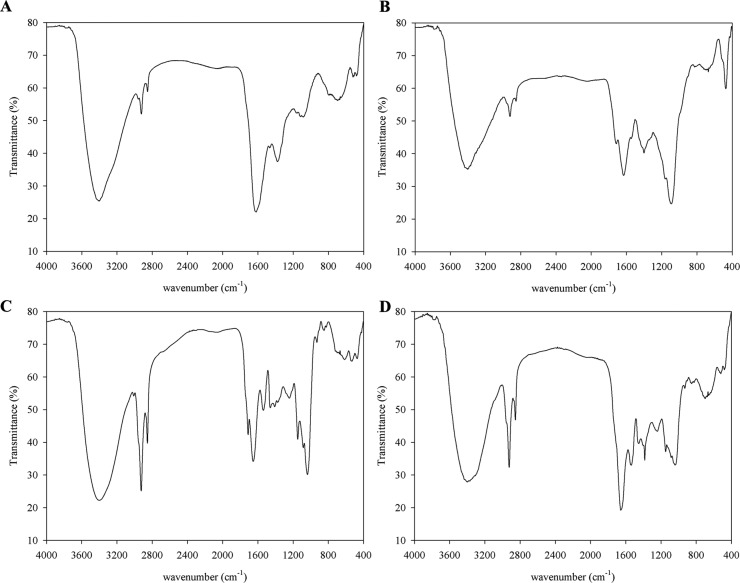
infrared spectra of commercial melanin (A), supernatant melanin (B), ghost melanin (C), of melanin treated with NaOH (D) collected in the transmission mode (4000-400 cm^-1^).

*Results of FT-IR spectroscopy* - The infrared spectra of the melanin samples are presented in Fig. 1 A-D. All samples of melanin showed a strong band at 3401 cm^-1^ corresponding to the primary amine (-NH_2_) or associated -OH; the other two bands at 2923 and 2852 cm^-1^ corresponded to -CH_3_ and -CH_2_ of the aliphatic structure. These bands agree with those found in the literature for other melanin samples (Alviano et al. 1991, Selvakumar et al. 2008, Kumar et al. 2011). The band at 1711 cm^-1^ observed in ghost melanin (Fig. 1C) corresponded to the aliphatic ketone (-C = O). The bands at 1654 cm^-1^ (ghost melanin and melanin treated with NaOH) (Fig. 1C-D); 1632 cm^-1^ (melanin supernatant) (Fig. 1B), and 1620 cm^-1^ (commercial melanin) (Fig. 1A) corresponded to C = C. Finally, the bands at 1089 and 1037 cm^-1^ present in the ghost melanin and melanin supernatant, respectively, corresponded to C-O.

**Fig. 2:**
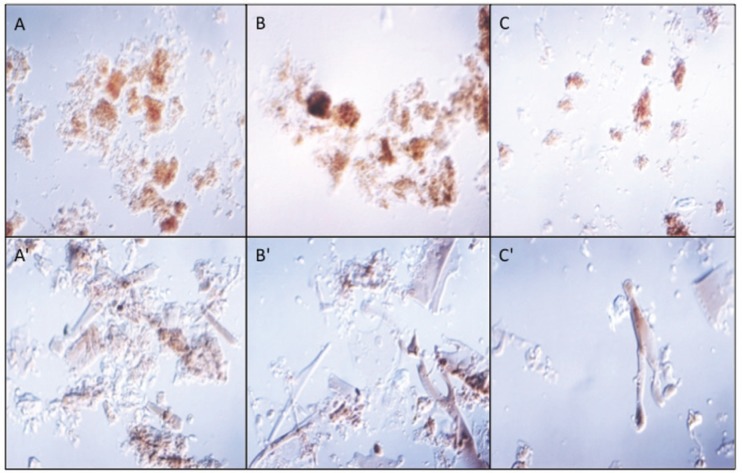
agglutination tests for detecting guinea pig antibodies to *Fonsecaea pedrosoi* melanin. (A) Melanin and (A') melanin ghost particles mixed with 1/256 immune serum from animal 1; (B) melanin and (B') melanin ghost particles mixed with 1/128 immune serum from animal 2; (C) melanin and (C') melanin ghost particles mixed with 1/128 pre-immune serum from animal 2.

*Guinea pig antibodies to F. pedrosoi melanin* - An agglutination test was conducted using pre-immunised guinea pig serum to show that non-specific antibodies did not detect agglutinate melanin or melanin ghost particles (Fig. 2C, C'), also that hydrophobicity was not sufficient to aggregate both melanin samples suspended in PBS under optical microscopy in the absence of serum. However, when melanin and melanin ghost particles were incubated with immunised serum, they clearly aggregated, indicating an antigen-antibody reaction even at high serum dilutions (Fig. 2).

*Complement activation* - The complement consumption percentage of melanin extracted from *F. pedrosoi* after incubation with serum chelated or not with magnesium EGTA was evaluated. Zymosan was used as a positive control of human complement activation *in vitro*, as this particle activates the complement system mainly through the alternative pathway and shows consumption of 100% of complement activity (Table). Normal serum was used as a negative control and presented consumption of only 1.2% of complement (Table).

**TABLE:** Complement consumption percentage of melanin extracted from *Fonsecaea pedrosoi* after incubation with serum chelated or not with magnesium EGTA

Samples	Serum + EGTA-Mg^2+^	Normal serum
Melanin particles^a^	61,07 ± 2,82%	82,67 ± 2,08%
Sensitized melanin particles^b^	64,18 ± 4,89%	100%
Melanin ghosts^c^	80,33 ± 1,97%	96,04 ± 1,13%
Sensitised melanin ghosts^b^	83,69 ± 2,43%	100%
Normal serum^d^	1,23 ± 0,46%	1,26 ± 0,60%
Zymosan^e^	100%	100%

a acid-basic extraction;

b 1/100 guinea pig anti-melanin;

c Novozym 234 extraction;

d negative control;

e positive control.

Our results showed that unsensitised melanin particles and melanin ghosts presented a complement consumption of 82.67 ± 2.08% and 96.04 ± 1.13%, respectively, when incubated in normal serum. When incubated with serum pre-treated with EGTA-Mg^2+^, which prevents activation of the classical pathway by chelating Ca^2+^, consumption of complement activity of approximately 61.07 ± 2.82% and 80.33 ± 1.92%, respectively, was observed. Sensitised melanin particles and melanin ghost showed improved complement consumption after activation, and both samples showed 100% complement consumption when sensitised (Table). Such sensitised particles did not show improved complement consumption in serum treated with EGTA-Mg^2+^. Reduced complement activity by melanin particles, melanin ghost, or positive control in the serum treated with EDTA was not observed (data not shown).

**Fig. 3:**
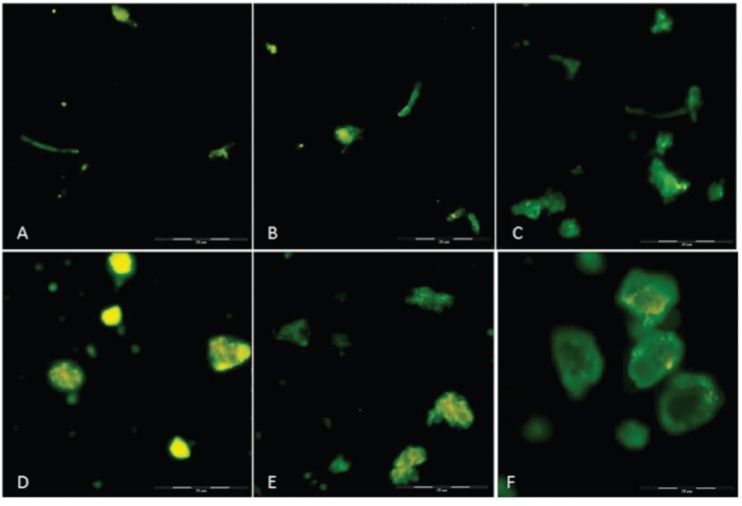
immunofluorescence for detecting C3 fragments on the surface of melanin particles and ghosts extracted from *Fonsecaea pedrosoi*. (A) Melanin ghost incubated with serum treated with EGTA-Mg^2+^, (B) melanin ghost without chelators, (C) melanin ghost sensitised with anti-melanin antibodies tested against serum without chelators, (D) melanin particles treated with EGTA-Mg^2+^, (E) melanin particles without chelators (F) melanin particles sensitised with anti-melanin antibodies tested against serum without chelators. Whole bar indicates 100 mm, divided into five 20-mm smaller bars.

*Direct immunofluorescence assay* - Deposition of serum C3 fragments on the sample surfaces was examined by immunofluorescence microscopy. As shown in Fig. 3, a similar pattern and intensity for C3 fragments was observed on the surface of both melanin ghosts (Fig. 3A-C) and melanin particles (Fig. 3D-F) when the samples were incubated in serum containing EGTA-Mg^2+^ (Fig. 3A, D) or not (Fig. 3B, E). Intense deposition of C3 was also observed on both sensitised melanin particles and melanin ghosts (Fig. 3C, F). No immunofluorescence was observed on melanin or melanin ghost particles when the complement activation used serum treated with EDTA (data not shown).

**Fig. 4:**
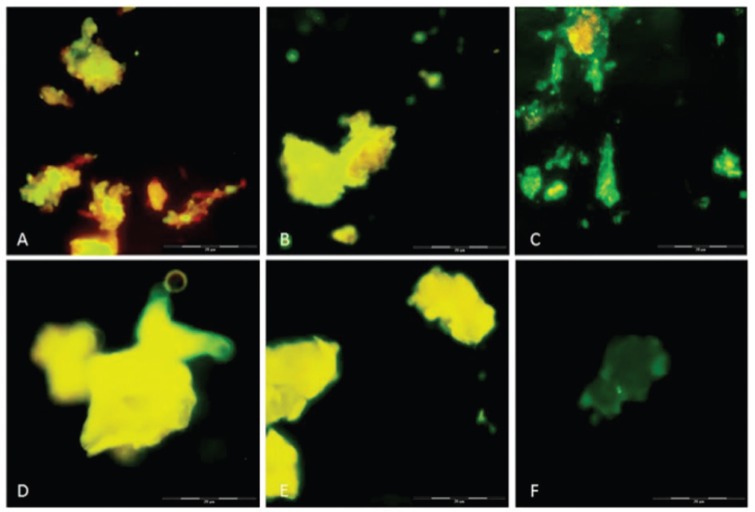
immunofluorescence for detecting C4 fragments on the surface of melanin particles and ghosts extracted from *F. pedrosoi*. (A) Melanin ghost incubated with serum treated with EGTA-Mg^2+^, (B) melanin ghost without chelators, (C) melanin ghost sensitised with anti-melanin antibodies tested against serum without chelators, (D) melanin particles treated with EGTA-Mg^2+^, (E) melanin particles without chelators, (F) melanin particles sensitised with anti-melanin antibodies tested against serum without chelators. Whole bar indicates 100 mm, divided into five 20-mm smaller bars.

*Indirect immunofluorescence assay* - Deposition of C4b onto the sample surfaces was examined by immunofluorescence microscopy. Fig. 4 shows that the component tested was found on the surface of both melanin ghosts (Fig. 4A-C) and melanin particles (Fig. 4D-F) when incubated with (Fig. 4A, D) or without EGTA-Mg^2+^ (Fig. 4B, D). C4b deposits were also observed on both sensitised melanin particles and melanin ghosts (Fig. 2C, F) and appeared to show stronger fluorescence in all cases compared to unsensitised samples. No immunofluorescence was observed on melanin or melanin ghost particles when the complement activation used serum treated with EDTA (data not shown).

**Fig. 5:**
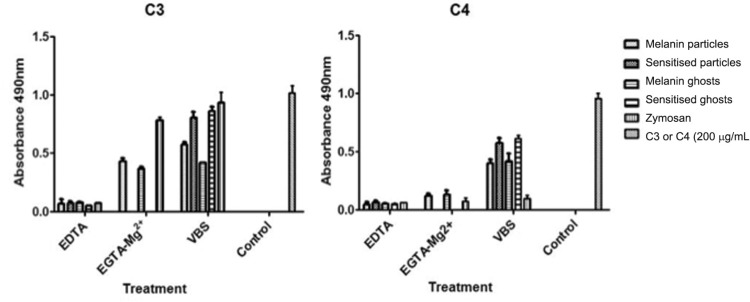
deposition of C3 and C4 fragments onto melanin samples and zymosan by enzyme-linked immunosorbent assay (ELISA). Antibodies against each component were added and bound antibodies were detected with a peroxidase conjugated secondary antibody. Values are optical densities (OD) measured at 490 nm. The OD values of the controls were subtracted from the values shown.

**Figure 6:**
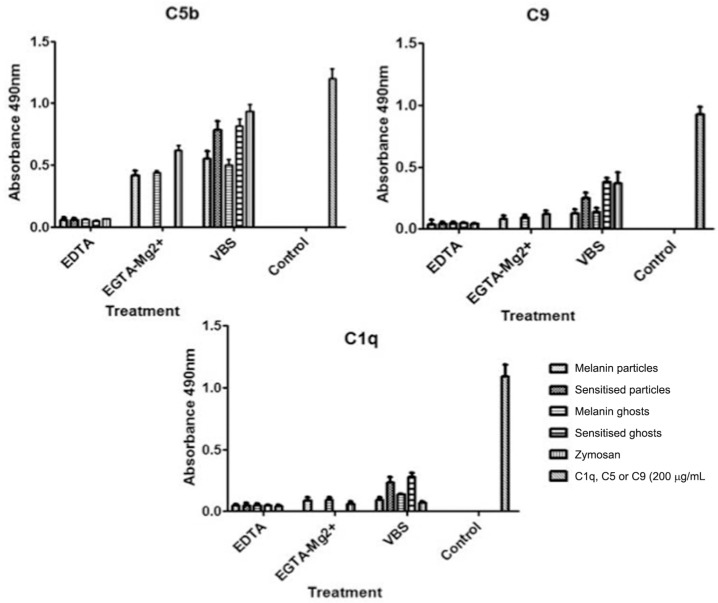
Deposition of C1q, C5, and C9 onto melanin samples and zymosan analysed by enzyme-linked immunosorbent assay (ELISA). Antibodies against each component were added and bound antibodies were detected with a peroxidase conjugated secondary antibody. Values are optical densities (OD) measured at 490 nm. The OD values of the controls were subtracted from the values shown.

*ELISA* - Complement component deposition onto the samples, after incubation in human serum, was further assayed by ELISA (Fig. 5). C3 fragments bound to both melanin particles and ghosts in the experiments using human serum treated or not with EGTA-Mg^2+^, confirming the pattern previously observed by microscopy (Fig. 5). As described above, melanin sensitisation with antibodies increased the number of bound C3 fragments (Fig. 5). Assays to detect C4 fragments on the surface of melanin samples showed that a small amount bound to their surface in the presence of EGTA-Mg^2+^, which supports the absence of the classical pathway. When samples were incubated with serum without chelators, the amount of C4 fragments bound doubled (Fig. 5). Sensitised melanin samples also showed a significant increase in bound C4 fragments (Fig. 5). C5b was detected on all melanin samples, sensitised or not, either in the presence or absence of EGTA-Mg^2+^ (Fig. 6). For C9 detection, a small amount bound to the unsensitised samples. However, sensitised melanin showed a noticeable improvement in the amount of this component (Fig. 6). A very small amount of bound C1q was detected on both melanin particles and ghost, while a considerable increase was observed in sensitised melanin samples (Fig. 6).

## DISCUSSION

Melanin pigment has been implicated in the pathogenesis of several microbial infections and is important for virulence. Previous studies demonstrated that melanin has antigenic and anti-inflammatory properties (Rosas et al. 2002). These findings led us to explore the interaction of *F. pedrosoi* melanin and the immune system. In a previous study, we demonstrated that the black fungus *F. pedrosoi* activated the complement system in human serum *in vitro* (mainly via the alternative pathway). Comparison of highly pigmented and hypopigmented fungal cells revealed that complement consumption was higher when pigmented mycelia were used (Pinto et al. 2011).

To confirm that the previous effect was related to melanin and not to others components exposed on the fungal cell surface, we extracted melanin particles and melanin ghosts from *F. pedrosoi* pigmented cells and evaluated their effects on complement activation. Data extracted from complement consumption by melanin particle or ghost (Table) illustrated that melanin elicited the complement mainly via an alternative pathway.

When samples were incubated in the serum not treated with chelators, increased complement consumption was observed (Table). Nevertheless, the results suggest mainly alternative pathway activation. Our data also show that, in all cases, the amount of C4 (Fig. 5) fragment deposited was considerably smaller on samples incubated with serum chelated with EGTA-Mg^2+^ than without chelators, supporting that the alternative pathway is mainly responsible for activation under these conditions. Because consumption was higher without the addition of EGTA, the difference observed may be related to classical pathway activation. As serum was previously absorbed with melanin and sheep erythrocytes, it did not contain specific antibodies that could activate the classical pathway by an antigen/antibody complex. However, the participation of the classical pathway by the action of acute phase proteins cannot be excluded (Gupta and Surolia 2004). Another possible explanation for this increased consumption is the presence of mannose binding lectin (MBL) and MBL-associated serine proteases (MASPs) in human serum (Selander et al. 2006). Together, MBL can recognise mannose patterns on the microorganism surface and MASPs can activate the system via the lectin pathway. The presence of rhamnose, mannose, galactose, and glucose residues in the melanin moiety may trigger the lectin pathway (Alviano et al. 1991).

Immunofluorescence analyses demonstrated the deposition of C3 and C4 fragments on the different melanin samples used in this study (Figs 3-4). Because Evan's blue dye causes non-fluorescent structures to emit red fluorescence, the samples can show three possible outcomes: red colour for non-reactant structures, yellow colour for few reactant structures, and greener as the number of reactions increases. The tested melanin samples showed that deposition occurred on most of the pigment surface.

Non-specific deposition was not examined because of the lack of C3 and C4 fragment detection onto melanin and melanin ghost after complement activation with serum treated with EDTA (data not shown). EDTA inhibits all complement activation pathways but does not inhibit non-specific deposition of serum proteins such as complement components under the conditions in this study. Additionally, all samples including zymosan were washed three times with PBS-Tween before the immunofluorescence or ELISA assays, and thus complement components were present because of covalent binding, reflecting actual complement activation.

The results of Torinuki et al. (1984) using sclerotic cells of *F. pedrosoi* also suggested that the activation and conversion of C3, analysed by immunoelectrophoresis, occurs entirely via the alternative pathway, as complement activation was not affected when the classical pathway was blocked by EGTA-Mg^2+^. Rosas et al. (2002) obtained similar results using melanin ghosts from *C. neoformans* and *Aspergillus niger.*

The ELISA results demonstrate that C3b, C4b, and C5b was present on the fungus, while C9 was very limited (Fig. 3). These results indicate that in the presence of human serum, the fungus activates and develops the cascade of complement activation but not to completion. Nonetheless, melanin's ability to bind C3b and C5b after complement activation may be important in spreading *F. pedrosoi* in the host. This indicates the formation of C3a and C5a, which can in turn function as chemoattractants for neutrophils and dendritic cells (Gutzmer et al. 2006). C5a is also involved in the activation of phagocytic cells, release of granule-based enzymes, and generation of oxidants (Guo and Ward 2005). Neutrophils, for example, recognise cells coated with C3 fragments (C3b and iC3b) through their complement receptors (CR3 and CR4 which bind iC3b or CR1 which binds to C3b) (Ricklin et al. 2010) as well as to the C5a anaphylatoxin (Guo and Ward 2005). The ability of melanin to quench free radicals is thought to reduce the susceptibility of fungal cells to oxidative damage (Nosanchuk and Casadevall 2006, Cunha et al. 2010), a fungal mechanism enabling escape from host immune defence processes (Chai et al. 2009).

Notably, *F. pedrosoi* secretes melanin during neutrophil infection (Rozental et al. 1996). Melanin fungal ghosts are also observed during the infection because even when the host cell successfully kills the fungus, melanin ghosts are not digested and can persist for a long time during infection (Nosanchuk et al. 2000). Sera from patients with chromoblastomycosis reacted with melanin secreted into the culture medium during fungal growth, suggesting that melanin synthesis also occurs *in vivo* during infection (Alviano et al. 2004).

We showed that melanin sensitisation with anti-melanin antibodies elicited full complement consumption (Table), indicating that through specific antibodies, the classical pathway is activated, greatly enhancing the complement response to this pigment. All results from sensitised samples showed improvement compared to unsensitised tests (Figs 3-6). C1q (Fig. 6) also strongly indicates classical pathway activation in these situations, as agglutination was observed when higher concentrations of antibodies were added (data not shown). However, C3 deposition is typically much faster by the classical pathway compared to the alternative pathway (Kozel 1998), indicating that its usage is much more valuable than the alternative. In contrast, to use the classical pathway, IgG or IgM is often necessary (Gadjeva et al. 2008). Adaptive immunity inclusion is typically necessary to obtain these antibodies. Thus, responses towards the antigen may be delayed for some time. For this reason, alternative pathway activation is crucial, as it contributes to antigen presentation to phagocytes, and therefore induces an adaptive immunity response, particularly Th17 lymphocytes (Wüthrich et al. 2015).

We found that melanin particles and ghosts extracted from *F. pedrosoi* activate the complement system, mainly via the alternative pathway. Additionally, melanin present on the cell surface of this fungus directly influences the activation, acting as an acceptor for complement proteins. C5b ensures anaphylatoxin formation and thus results in chemotaxis and activation of polymorphonuclear neutrophils. Indirectly, melanin may promote pathogen internalisation by phagocytic cells via complement receptors (CR1, CR3, CR4) as a strategy to allow their persistence on the host, as melanin inhibits nitric oxide production (Cunha et al. 2010). Furthermore, the presence of C1q and C9 indicate that the activation process reaches its full potential from the initial steps to membrane attack complex formation when the classical pathway is involved. However, deposited C9 cannot form intermembrane canals on *F. pedrosoi* because of the cell wall thickness (Santos et al. 2007).

The complement system is an important branch of innate immunity, and its activation by *F. pedrosoi* melanin may be important in the balance of the host cell response and progress of infection and fungal elimination.

## References

[B1] Alviano CS, Farbiarz SR, de Souza W, Angluster J, Travassos LR (1991). Characterization of *Fonsecaea pedrosoi* melanin. J Gen Microbiol.

[B2] Alviano DS, Franzen AJ, Travassos LR, Holandino C, Rozental S, Ejzemberg (2004). Melanin from *Fonsecaea pedrosoi* induces production of human antifungal antibodies and enhances the antimicrobial efficacy of phagocytes. Infect Immun.

[B3] Chai LYA, Netea MG, Vonk AG, Kullberg B (2009). Fungal strategies for overcoming host innate immune response. Med Mycol.

[B4] Correia RT, Valente NY, Criado PR, Martins JE (2010). Chromoblastomycosis: study of 27 cases and review of medical literature. An Bras Dermatol.

[B5] Cunha MM, Franzen AJ, Alviano SA, Zanardi E, Alviano CS, de Souza W (2005). Inhibition of melanina synthesis pathway by tricycazole increases susceptibility of *Fonsecaea pedrosoi* against mouse macrophages. Microsc Res Tech.

[B6] Cunha MM, Franzen AJ, Seabra SH, Herbst MH, Vugman NV, Borba LP (2010). Melanin in *Fonsecaea pedrosoi*: a trap for oxidative radicals. BMC Microbiol.

[B7] Gadjeva MG, Rouseva MM, Zlatarova AS, Reid KB, Kishore U, Kojouharova MS (2008). Interaction of human C1q with IgG and IgM: revisited. Biochemistry.

[B8] Gómez BL, Nosanchuk JD (2003). Melanin and fungi. Curr Opin Infect Dis.

[B9] Granja LF, Pinto L, Almeida CA, Alviano DS, da Silva MH, Ejzemberg R (2010). Spores of *Mucor ramosissimus*, *Mucor plumbeus* and *Mucor circinelloides* and their ability to activate human complement system *in vitro*. Med Mycol.

[B10] Guo RF, Ward PA (2005). Role of C5a in inflammatory responses. Annu Rev Immunol.

[B11] Gupta G, Surolia A (2004). Collectins: sentinels of innate immunity. Bioessays.

[B12] Gutzmer R, Köther B, Zwirner J, Dijkstra D, Purwar R, Wittmann M (2006). Human plasmacytoid dendritic cells express receptors for anaphylatoxins C3a and C5a and are chemoattracted to C3a and C5a. J Invest Dermatol.

[B13] Kozel TR (1998). Complement activation by pathogenic fungi. Res Immunol.

[B14] Kumar CG, Mongolla P, Pombala S, Kamle A, Joseph J (2011). Physicochemical characterization and antioxidant activity of melanin from a novel strain of *Aspergillus bridgeri* ICTF-201. Lett Appl Microbiol.

[B15] Nosanchuk JD, Casadevall A (2006). Impact of melanin on microbial virulence and clinical resistance to antimicrobial components. Antimicrob Agents Chemother.

[B16] Nosanchuk JD, Rosas AL, Lee SC, Casadevall A (2000). Melanisation of *Cryptococcus neoformans* in human brain tissue. Lancet.

[B17] Pinto L, Granja LF, Alviano DS, da Silva MH, Alviano CS, Ejzemberg R (2011). Activation of the human complement system by pigmented and hypopigmented mycelia of the fungus *Fonsecaea pedrosoi*. Mycoses.

[B18] Ricklin D, Hajishengallis G, Yang K, Lambris JD (2010). Complement - a key system for immune surveillance and homeostasis. Nat Immunol.

[B19] Romero-Martinez R, Wheeler M, Guerrero-Plata A, Rico G, Torres-Guerrero H (2000). Biosynthesis and functions of melanin in *Sporothrix schenckii*. Infect Immun.

[B20] Rosas AL, MacGill RS, Nosanchuk JD, Kozel TR, Casadevall A (2002). Activation of the alternative complement pathway by fungal melanins. Clin Diagn Lab Immunol.

[B21] Rosas AL, Nosanchuk JD, Gomez BL, Edens WA, Henson JM, Casadevall A (2000). Isolation and serological analyses of fungal melanins. J Immunol Methods.

[B22] Rozental S, Alviano CS, Souza W de (1996). Fine structure and cytochemical study of the interaction between *Fonsecaea pedrosoi* and rat polymorphonuclear leukocyte. J Med Vet Mycol.

[B23] Runza VL, Schwaeble W, Männel DN (2008). Ficolins: novel pattern recognition molecules of the innate immune response. Immunobiology.

[B24] Santos ALS, Palmeira VF, Rozental S, Kneipp LF, Nimrichter L, Alviano DS (2007). Biology and pathogenesis of Fonsecaea pedrosoi, the major etiologic agent of chromoblastomycosis. FEMS Microbiol Rev.

[B25] Selander B, Mårtensson U, Weintraub A, Holmström E, Matsushita M, Thiel S (2006). Mannan-binding lectin activates C3 and the alternative complement pathway without involvement of C2. J Clin Invest.

[B26] Selvakumar P, Rajasekar S, Periasamy K, Raaman N (2008). Isolation and characterization of melanin pigment from Pleurotus cystidiosus (telomorph of Antromycopsis macrocarpa). World J Microbiol Biotechnol.

[B27] Speth C, Rambach G, Würzner R, Lass-Flörl C (2008). Complement and fungal pathogens: an update. Mycoses.

[B28] Torinuki W, Okohchi K, Takematsu H, Tagami H (1984). Activation of the alternative complement pathway by *Fonsecaea pedrosoi*. J Invest Dermatol.

[B29] Torres-Guerrero E, Isa-Isa R, Isa M, Arenas R (2012). Chromoblastomycosis. Clin Dermatol.

[B30] Wüthrich M, Wang H, Li M, Lerksuthirat T, Hardison SE, Brown GD (2015). *Fonsecaea pedrosoi* - induced Th17-cell differentiation in mice is fostered by Dectin-2 and suppressed by Mincle recognition. Eur J Immunol.

